# Cardiopulmonary and Muscular Interactions: Potential Implications for Exercise (In)tolerance in Symptomatic Smokers Without Chronic Obstructive Pulmonary Disease

**DOI:** 10.3389/fphys.2019.00859

**Published:** 2019-07-10

**Authors:** Paulo de Tarso Muller, Gisele Walter Barbosa, Denis E. O’Donnell, J Alberto Neder

**Affiliations:** ^1^Laboratory of Respiratory Pathophysiology, Respiratory Division, Department of Medicine, Federal University of Mato Grosso do Sul, Campo Grande, Brazil; ^2^Laboratory of Clinical Exercise Physiology, Respiratory Investigation Unit, Division of Respiratory and Critical Care Medicine, Department of Medicine, Queen’s University, Kingston, ON, Canada

**Keywords:** smoking, exercise intolerance, physical activity, dyspnea, Fatigue

## Abstract

Smoking and physical inactivity are important preventable causes of disability and early death worldwide. Reduced exercise tolerance has been described in smokers, even in those who do not fulfill the extant physiological criteria for chronic obstructive pulmonary disease (COPD) and are not particularly sedentary. In this context, it is widely accepted that exercise capacity depends on complex cardio-pulmonary interactions which support oxygen (O_2_) delivery to muscle mitochondria. Although peripheral muscular factors, O_2_ transport disturbances (including the effects of increased carboxyhemoglobin) and autonomic nervous system unbalance have been emphasized, other derangements have been more recently described, including early microscopic emphysema, pulmonary microvascular disease, ventilatory and gas exchange inefficiency, and left ventricular diastolic dysfunction. Using an integrative physiological approach, the present review summarizes the recent advances in knowledge on the effects of smoking on the lung-heart-muscle axis under the stress of exercise. Special attention is given to the mechanisms connecting physiological abnormalities such as early cardio-pulmonary derangements, inadequate oxygen delivery and utilization, and generalized bioenergetic disturbances at the muscular level with the negative sensations (sense of heightened muscle effort and breathlessness) that may decrease the tolerance of smokers to physical exercise. A deeper understanding of the systemic effects of smoking in subjects who did not (yet) show evidences of COPD and ischemic heart disease – two devastating smoking related diseases – might prove instrumental to fight their ever-growing burden.

“So marked is the effect of tobacco in relaxing the whole of the muscular system, that before the days of chloroform it was employed in surgical operation, in which it was necessary that the muscles should be perfectly cleaned.”

*Sir Morell Mackenzie, in: The Tobacco Habit. Its History and Pathology. Herbert H. Tidswell, London, J&H Churchil, 1912.*

## Introduction

Cigarette smoking, the most important preventable cause of death worldwide, is strongly associated with the poor quality of life and health-care resources utilization. (World Health Organization, 2011) Physical inactivity, a common finding in smokers, has also been mechanistically linked to a plethora of nontransmissible diseases (World Health Organization, 2010; Lee et al., 2012). There is, therefore, increasing awareness of the link between exercise intolerance and smoking (Clini et al., 2016); moreover, the last decades witnessed a growing debate on the consequences of preclinical chronic obstructive pulmonary disease (COPD) – the prototype of a smoking-related disease – on clinical outcomes, including exercise intolerance (Caram et al., 2016; Chen et al., 2016; Rhee et al., 2017; Soriano et al., 2018).

In this context, reduced maximal and submaximal exercise tolerance and breathlessness on daily life (modified Medical Research Council dyspnea score, ≥2) have been described in a subgroup of smokers, even when they do not fulfill the extant physiological criteria for COPD (Klein et al., 1992; Misigoj-Durakovic et al., 2012; Liu et al., 2015; Regan et al., 2015; Elbehairy et al., 2016, 2017a; Woodruff et al., 2016; Di Marco et al., 2017; Martinez et al., 2017; Walter Barbosa et al., 2017; Fuertes et al., 2018) and they are not particularly sedentary (Misigoj-Durakovic et al., 2012; Fuertes et al., 2018). It is widely accepted that exercise intolerance is the final result of abnormalities in the complex interaction between large systems (mainly pulmonary and cardiocirculatory) which support O_2_ delivery to muscle mitochondria (Burtscher, 2013; Gabriel and Zierath, 2017). In smokers, these abnormalities have been mainly ascribed to peripheral muscular factors (Wüst et al., 2008a,c; Degens et al., 2015; Al-Bashaireh et al., 2018; Nogueira et al., 2018). Oxygen (O_2_) transport disturbances [including high blood carboxy-hemoglobin levels (HbCO); Huie, 1996; Maehara et al., 1997; Bye et al., 2008; Barn et al., 2018] and, potentially, cardiocirculatory abnormalities (Gidding et al., 1995; Lauer et al., 1997; Bernaards et al., 2003; Kobayashi et al., 2004; Tello et al., 2005; Papathanasiou et al., 2007), and autonomic nervous system unbalance (Kotamäki, 1995; Mendonca et al., 2011; Ide and Tabira, 2013). Of note, influential reviews on the topic (Huie, 1996; Heishman et al., 2010) did not consider the modulatory effects of abnormalities that only recently have gained more attention, including early emphysema (Sashidhar et al., 2002; Fain et al., 2006; Grydeland et al., 2009; Harris et al., 2012; Mohamed Hoesein et al., 2014; Regan et al., 2015; Alcaide et al., 2017; Crossley et al., 2018), pulmonary microvascular disease (Nana-Sinkam et al., 2007; Schweitzer et al., 2011; Harris et al., 2012; Estépar et al., 2013; Schmekel et al., 2013; Iyer et al., 2016; Rizzi et al., 2016; Aaron et al., 2017, 2018; Saruya et al., 2017), ventilatory inefficiency, (Gläser et al., 2011; Elbehairy et al., 2016; Walter Barbosa et al., 2017), gas exchange abnormalities (Gläser et al., 2010, 2011, 2013; Elbehairy et al., 2015), and left ventricular diastolic dysfunction (Tello et al., 2005; Payne et al., 2006; Gulel et al., 2007; Yilmaz et al., 2007; Bennet et al., 2010; Talukder et al., 2011; Leary, 2016; Nadruz et al., 2016).

**Figure 1:**
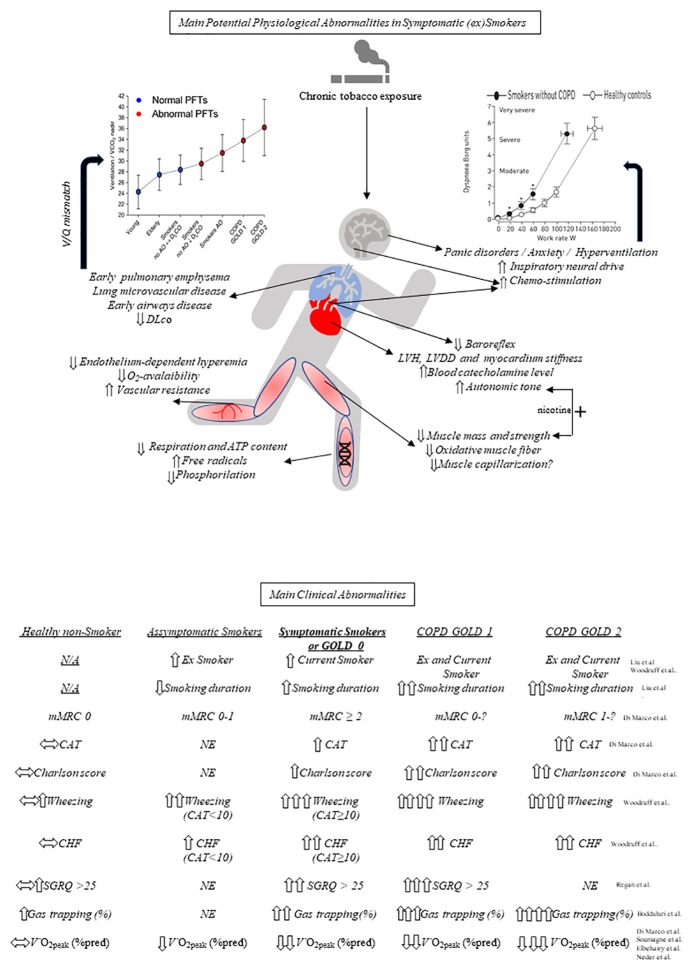
Potential mechanisms underlying exercise (in)tolerance in symptomatic smokers and comparative main clinical abnormalities. Above, key respiratory, cardiocirculatory, and peripheral muscular (including mithocondrial) effects of chronic cigarette smoking are highlighted. Ventilation (V)/perfusion (Q) mismatch, in particular, may increase the ventilatory demand to exercise (*left graph*, reprinted with permission of the American Thoracic Society. Annals ATS. Vol. 14, No. Supplement_1, Jul 01, 2017) which, in association, with other source of ventilatory stimuli (and potential psychological/behavioral traits) increases exertional dyspnea at a given work rate (*right graph,* reproduced with permission of the ©ERS (2019): Elbehairy et al., 2016). Below, main characteristic clinical abnormalities of symptomatic (ex)smokers compared with asymptomatic smokers, healthy nonsmokers, COPD GOLD 1 and COPD GOLD 2. See text for further elaboration. PFT, pulmonary function tests; AO, airways obstruction; GOLD, global initiative for obstructive lung disease; COPD, chronic obstructive pulmonary disease; CO, carbon monoxide; DLco, lung diffusion capacity for CO; ATP, adenosine triphosphate; LVH, left ventricular hypertrophy; LVDD, left ventricular diastolic dysfunction; N/A, not applicable; mMRC, modified Medical Research Council; CAT, COPD Assessment Test; NE, not evaluated; CHF, chronic heart failure; SGRQ, St George’s Respiratory Questionnaire; *V*′O_2peak_, peak oxygen consumption; ^*^p < 0.05 at similar work-rate.

As smoking duration is the main trigger for symptoms and risk for COPD, (Liu et al., 2015; Bhatt et al., 2018), it is conceivable that there is a continuum from reduced maximal exercise capacity at a young age toward exercise intolerance in middle-age and older smokers. In fact, some smokers do experience a larger-than-expected (by aging) decrease in maximal O_2_ consumption (V′O_2max_) (0.2–0.5 mL × min^−1^ × kg^−1^/year) (Burtscher, 2013; Roman et al., 2016; Rodrigues et al., 2018), an effect that might be, at least in part, genetically determined (Ross et al., 2018). Thus, there is growing evidence in favor of derangements in the muscle-lung-heart axis (Bye et al., 2008; Grydeland et al., 2009; Gläser et al., 2013; Degens et al., 2015; Regan et al., 2015; Leary, 2016; Alcaide et al., 2017; Saruya et al., 2017; Walter Barbosa et al., 2017; Barn et al., 2018) that may have sensory consequences and contribute to poor exercise tolerance in ever-smokers or former smokers without COPD (Figure 1, above). Overall, these derangements are followed by clinical traits increasingly described in the medical literature (Figure 1, below).

The present review aims to succinctly summarize the recent advances in our knowledge on the effects of smoking on the muscle-lung-heart axis under the stress of exercise. Despite the fact that there is an acute-on-chronic effect of current smoking on these interactions, we will refrain from discussing the large body of clinical and experimental evidence showing the deleterious effects of acute tobacco smoking in nicotine-naive subjects (Johnston et al., 2018). Thus, we will focus on the chronic consequences of smoking on exercise intolerance from an integrative physiological perspective, giving special attention to the ancillary effects of aging, and physical inactivity.

## Peripheral Muscular Abnormalities

Despite its relevance to exercise intolerance in subjects with COPD (Aliverti and Macklem, 2008; Debigaré and Maltais, 2008), there is a lack of in-depth discussion about the effects of smoking on the “muscle-mitochondria compartment” as a potential limiting factor in non-COPD smokers. In any case, reduced muscle strength and/or mass have been described in smokers (Montes de Oca et al., 2008; Wüst et al., 2008b, 2009; Kok et al., 2012; Rom et al., 2012; Degens et al., 2015); of note, two meta-analyses suggested an independent effect (from physical inactivity) of cigarette smoking on reducing muscle mass (Rom et al., 2012; Steffl et al., 2015). Conversely, chronic sympathetic nerve over-excitation induced by nicotine may counterbalance the potential deleterious effects of smoking on muscle mass (Mündel and Jones, 2006). Muscle wasting after chronic tobacco exposure in some smokers might be related to increased ubiquitin-mediated proteolysis (Petersen et al., 2007; Liu et al., 2011; Rom et al., 2012). In addition, smoking may inhibit anabolic pathways and protein synthesis in the quadriceps (Degens et al., 2015; Madani et al., 2018) (as extensively reviewed in Degens et al., 2015). Current and former experimental data support changes in muscle fiber endotype toward a less oxidative profile (Orlander et al., 1979; Montes de Oca et al., 2008; Krüger et al., 2015, 2018). However, reduced muscle capillarization remain questionable (Montes de Oca et al., 2008; Wüst et al., 2008b; Nogueira et al., 2018). At the subcellular level, mitochondrial DNA might be damaged in smokers without COPD (Fetterman et al., 2017).

These structural changes might negatively impact on muscle bioenergetics and metabolism. Spillover of inflammatory mediators produced by lung epithelial cells in response to smoking may reach the striated muscles with negative bioenergetic consequences (Fetterman et al., 2017; Madani et al., 2018). The mitochondrion, as important source of biochemical and thermal energy, is a key target for smoking toxicity, leading to reduced respiration, decreased ATP content, and increased production of free radicals in a dose- and time-dependent manner (Neves et al., 2016; Fetterman et al., 2017; Madani et al., 2018). As a consequence, smokers may present with impaired oxidative phosphorylation (as extensively reviewed by Fetterman et al., 2017). Other metabolic derangements with a potential to impact on physical performance include: An appreciable (10%) increase in energy expenditure at rest compared to nonsmoking subjects (Hofstetter et al., 1986), impaired sarcoplasmic reticulum Ca^++^ uptake in myofibres (Nogueira et al., 2018), and impaired insulin-dependent glycogen recovery from exercise (Jensen et al., 1995). An early lactate threshold might be the final consequence of these bioenergetic derangements in association with chronically low levels of muscle activation, i.e., sedentarism (Miyatake et al., 2011; Lauria et al., 2017).

However, only a few studies showed reduced peripheral muscle strength (Kok et al., 2012) or reduced fatigue resistance to electrical stimulation under controlled conditions in smokers compared with nonsmokers with similar physical (in)activity scores (Wüst et al., 2008b,c). In fact, this is a major confounder as several studies failed to show lower scores of peripheral muscle fatigue (Orlander et al., 1979; Larsson and Orlander, 1984) or force generation (Wüst et al., 2008b,c) in smokers when compared to controls paired by self-reported physical activity. Diminished resistance to fatigue in smokers was demonstrated using effort-independent techniques, such as electrically evoked muscle contractions (Wüst et al., 2008c) and CO inhalation (Morse et al., 2008). Interestingly, however, nicotine may also have ergogenic effects through augmented release of adrenaline and enhanced performance of fast-twitch muscle fibers (Johnston et al., 2018). Thus, any potential increase in muscle fatigability in smokers might be compensated by the central excitatory actions of nicotine leading to preserved time to task failure compared to equally sedentary controls (Orlander et al., 1979; Larsson and Orlander, 1984).

## Cardiocirculatory Abnormalities

Large population-based studies found subtle cardiac structure and function abnormalities, which could be mechanistically related to smoking (Gidding et al., 1995; Lauer et al., 1997; Bernaards et al., 2003; Payne et al., 2006; Nadruz et al., 2016); of note, some of these studies suggested increased left ventricular mass and chronotropic incompetence during exercise (Gidding et al., 1995; Lauer et al., 1997; Payne et al., 2006). Key abnormalities found in chronic smokers include increased autonomic activity (Kotamäki, 1995; Lauer et al., 1997; Mendonca et al., 2011; Ide and Tabira, 2013), elevated catecholamine levels (Laustiola et al., 1988; Kotamäki, 1995; Chelland Campbell et al., 2008), acute and chronic rest systemic arterial hypertension, largely related with arterial stiffness and secondary to tobacco induced endothelial dysfunction (Scallan et al., 2010), altered exercise heart rate-systolic blood pressure product (Papathanasiou et al., 2007), left ventricular diastolic dysfunction (Tello et al., 2005; Payne et al., 2006; Gulel et al., 2007; Yilmaz et al., 2007; Bennet et al., 2010; Leary, 2016; Nadruz et al., 2016), and direct myocardial depression due to CO in heavy smokers (Bye et al., 2008). Of note, despite the fact that exposure to tobacco smoke is a strong risk factor for pulmonary hypertension and chronic thromboembolic disease, (Schiess et al., 2010; Weissmann et al., 2012; Keusch et al., 2014), there is a lack of studies addressing potential abnormalities in pulmonary vascular conductance during exercise in symptomatic non-COPD smokers. Isolated left ventricular diastolic dysfunction was previously associated with reduced exercise capacity in some populations (Genovesi-Ebert et al., 1994; Barmeyer et al., 2009; Grewal et al., 2009). However, there was only a weak relationship between left ventricular dysfunction and tolerance to stress exercise testing in smokers without COPD (Grewal et al., 2009). Of note, we could not confirm these findings in patients with COPD (Muller et al., 2018). Downregulation of β-adrenoceptors (Laustiola et al., 1988) and blunted heart rate during exercise are described maladaptations to chronic smoking (Lauer et al., 1997). Conversely, resting heart rate is commonly increased, likely due to the combined effects of: the pharmacological action of nicotine (Turner and McNicol, 1993), increased circulating levels of catecholamine (Laustiola et al., 1988; Kotamäki, 1995; Chelland Campbell et al., 2008), modulatory effects on baroreflex function (Bernaards et al., 2003; Papathanasiou et al., 2007; Mendonca et al., 2011) and chronic reduction in the vagal drive (Mendonca et al., 2011). Thus, resting tachycardia, in association with myocardium stiffness (Gidding et al., 1995) and diastolic dysfunction, (Tello et al., 2005; Payne et al., 2006; Gulel et al., 2007; Yilmaz et al., 2007; Talukder et al., 2011) may critically interfere with the ideal diastolic time-pressure product necessary to optimize left ventricular filling (Fisher, 2014). In fact, O_2_-pulse – a surrogate for stroke volume under certain conditions – was found lower during submaximal exercise in smokers compared to nonsmoker controls (Kobayashi et al., 2004). In addition, Kimura et al. (2007) using near-infrared spectroscopy showed increased O_2_ extraction at the right vastus lateralis during incremental exercise testing in the majority of “healthy” smokers compared to nonsmokers. This is in line with potential decrements in muscle O_2_ delivery caused by central derangements.

Muscle hyperemia on exercise due to microcirculatory adaptations is highly dependent on shear stress to induce nitric oxide (NO) release, i.e., endothelium-dependent vascular relaxation (Green et al., 2017). Smoking-induced oxidative stress is a trigger for a generalized vascular inflammation (Golbidi et al., 2018; Madani et al., 2018), the latter being associated with: lower expression of endothelial NO synthetase, increased expression of TNF-α, IL-6, and IL-1β (Golbidi et al., 2018), downregulation of IL-10 (Allam et al., 2013), increased adhesion of inflammatory cells stimulated by ICAM-1 and IL-8 (Madani et al., 2018) and, ultimately, disruption of endothelial integrity as a protective barrier (Golbidi et al., 2018). These abnormalities may impair the endothelium-dependent hyperemic response to exercise (Barua et al., 2002) and increase arterial vascular resistance (Degens et al., 2015). It is noteworthy that impairment in endothelium-dependent hyperemia has been associated with lower exercise tolerance in smokers (Heffernan et al., 2010; Montero, 2015).

In addition to these cardiocirculatory abnormalities, muscle O_2_ delivery on exercise may be impaired due to the deleterious consequences of increased (HbCO) as CO has a ~ 250 higher affinity to Hb compared to O_2_ (Maehara et al., 1997; Kimura et al., 2007; Keramidas et al., 2012). Smokers may show up to 9% HbCO leading to decrements in O_2_ content similar to those found in hypoxemic patients (Degens et al., 2015). High levels (two to three times normal range) can persist up to 90 min after smoking (Jarvis et al., 1987). Accordingly, several animal- and human-based studies demonstrated the deleterious effects of HbCO on submaximal (Maehara et al., 1997; Keramidas et al., 2012) and maximal exercise capacity (Vogel and Gleser, 1972; Aronow and Cassidy, 1975; Aronow et al., 1977; Klausen et al., 1983). These findings should be tempered with others which failed to show alterations in endurance (Turner and McNicol, 1993; Ryan et al., 2016). These discrepancies might be linked to the large inter-study variability on CO exposure or individual differences in CO clearance (Zavorsky et al., 2012). Of note, exercise on room air accelerates CO elimination compared to resting and moderate exercise is as effective as breathing 100% O_2_ at rest on this regard (Zavorsky et al., 2012). The deleterious consequences of high (HbCO) might be particularly important in the presence of comorbidities: low-dose inhaled CO (Aronow, 1976) and nicotine patch in substitution to smoking (Mahmarian et al., 1997) have been implicated in lower exercise capacity seen in smokers with ischemic heart disease.

## Respiratory Abnormalities

Spirometrically occult airways and lung parenchymal disease, pulmonary microvascular disease, gas exchange, and respiratory muscle abnormalities could potentially contribute to decrease exercise tolerance due to exertional dyspnea in symptomatic smokers (Hamari et al., 2010; Estépar et al., 2013; Rennard and Drummond, 2015; Elbehairy et al., 2016; Woodruff et al., 2016; Bodduluri et al., 2017; Martinez et al., 2017; Bostanci et al., 2019). Airway disease with chronic bronchitic symptoms is largely recognized in smokers without COPD (Rennard and Drummond, 2015; Woodruff et al., 2016; Martinez et al., 2017). It is conceivable that dysfunction in cystic fibrosis transmembrane conductance regulator (CFTR) might be mechanistically involved in the chronic bronchitis seen in some smokers without COPD, thereby leading to a clinical phenotype similar to mild cystic fibrosis (Raju et al., 2016). There is limited evidence that these symptoms might be related to reduced daily physical activity, independent of age and sex (Woodruff et al., 2016). In a large observational study (SPIROMICS) (Martinez et al., 2017), imaging evidence of initial airway disease, more frequent exacerbations, and poorer exercise tolerance were found in symptomatic current or former smokers with normal pulmonary function compared to nonsmokers and asymptomatic smokers with airflow limitation. Of note, about 50% of smokers without airway obstruction have symptoms such as dyspnea (Regan et al., 2015). Symptomatic smokers with dyspnea and preserved lung function may present with abnormally increased airway wall thickening on high-resolution computerized tomography scans (HRCT), suggesting early involvement of the small airways (Regan et al., 2015; Woodruff et al., 2016). Interestingly, although airway wall thickening decreases with higher age, smokers maintain higher airway wall thickening throughout aging (Telenga et al., 2017). In fact, increased closure volume of the small airways and high peripheral airway resistance by impulse oscillometry might be seen in smokers with dyspnea on exertion (Di Marco et al., 2017). Incipient/mild emphysema can also be found in heavy smokers with preserved spirometry (Regan et al., 2015), being occasionally associated with poor exercise tolerance and increased self-reported activity limitation on daily life (Regan et al., 2015; Alcaide et al., 2017). Kirby et al. (2013) and Pike et al. (2015) using advanced magnetic resonance imaging (MRI) identified substantial ventilation inhomogeneity in ex-smokers without airflow limitation; of note, this was spatially coincident with incipient/mild emphysema demonstrated on CT.

**Figure 2:**
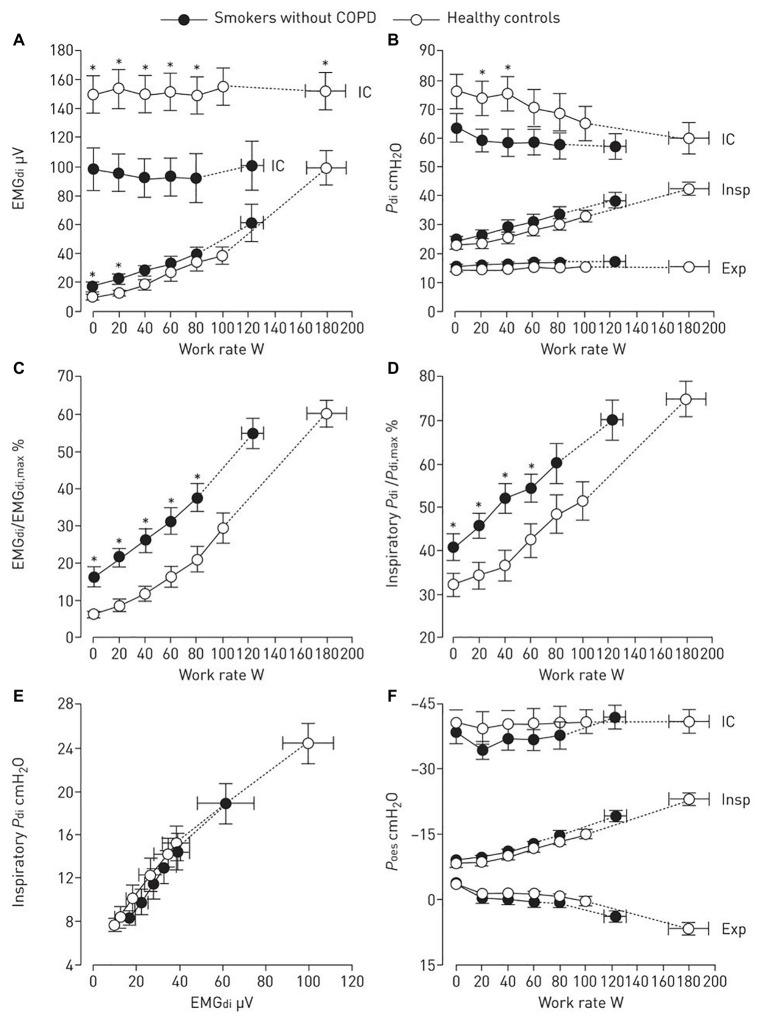
Diaphragm electromyography (EMG_di_, **A** and **C**), transdiaphragmatic pressure (*P*_di_, **B,D** and **E**), and oesophageal pressure (*P*_oes_, **F**) are shown during incremental cycle exercise in smokers without chronic obstructive pulmonary disease (COPD) and age-matched healthy controls. Dynamic maximal measurements during inspiratory capacity (IC) manoeuvres are also shown. (Reproduced with permission of the ©ERS 2019: Elbehairy et al., 2016). Data are presented as mean ± SEM. ^*^p < 0.05 smokers without COPD versus healthy controls at rest, at standardized work rates or at peak exercise. Insp, inspiratory; Exp, expiratory.

How those abnormalities could be mechanistically linked to activity-related dyspnea in smokers? Heightened awareness of increased efferent activity from bulbopontine and cortical motor centers to the inspiratory muscles are closely linked to exertional dyspnea (Ward et al., 2005). Elbehairy et al. (2016) found that higher fractional inspiratory neural drive to the diaphragm in smokers without COPD was secondary to compensatory increases in inspiratory diaphragm electromyographic activity to overcome increased airways resistance and lower maximal activation (Figure 2). Severe leg discomfort also contributed to exercise intolerance in this study: peripheral muscle weakness (Degens et al., 2015), greater motor command output (Ward et al., 2005; Elbehairy et al., 2016), and high perceived effort (relative to maximum) (Furlanetto et al., 2014) could be mechanistically involved in these findings (see also Peripheral Muscular Abnormalities section). Also, there are limited data pointing out for attenuated peripheral metaboreflex in non-COPD smokers (Drew et al., 2012). Of note, no study to date has specifically investigated whether symptomatic smokers without COPD may present with impaired respiratory muscle metaboreflex. If this is experimentally demonstrated, such results would provide the basis for additional studies exploring the hypothesis of blood flow redistribution from the locomotor muscles to the overburden respiratory muscles in these subjects (Oliveira et al., 2015).

It is also plausible that increased chemo-stimulation as a result of higher physiological dead space (increased V′E/V′CO_2_, ventilatory inefficiency) contributes to a higher inspiratory neural drive during tidal breathing in some smokers (Elbehairy et al., 2016; Weatherald et al., 2018). Increased V′E/V′CO_2_, likely reflecting high VD/VT, was found in smokers with only mild spirometric abnormalities (Elbehairy et al., 2017a,b) and smokers with low lung diffusion capacity for CO (Walter Barbosa et al., 2017). Importantly, a large populational study found that, after careful control for confounders, chronic cigarette smoking was associated with increased alveolar-arterial gradient and dead space on exercise (Gläser et al., 2010, 2013). Despite the absence of overt hypoxemia, there is evidence that smokers without COPD may present with large carotid bodies (Cramer et al., 2014; Tan et al., 2019), potentially increasing peripheral chemosensitivity and the inordinary ventilatory response to exercise found in some smokers. In any case, high dead space might reflect enlarged areas of increased ventilation/perfusion relationship independent of emphysema, i.e., early microvascular disease (Harris et al., 2012; Estépar et al., 2013; Saruya et al., 2017). Another piece of indirect evidence suggesting early pulmonary microvascular disease is the common finding of out-of-proportion decrease in DLco relative to macroscopic emphysema burden in symptomatic smokers (Kirby et al., 2013). It is also noteworthy that, at least in smoking rodent models, pulmonary vascular changes with neomuscularization of precapillary arteries may precede the development of emphysema (Ferrer et al., 2009, 2011) – as proposed by Liebow six decades ago (Liebow, 1959). Moreover, significant remodeling of the pulmonary arteries has been observed in heavy smokers (Santos et al., 2002). Smoking has been associated with endothelial (Schweitzer et al., 2011; Schmekel et al., 2013) and epithelial damage (Madani et al., 2018): cigarette smoke products may cause pulmonary vascular remodeling through either a direct effect on endothelial cells or an inflammatory mechanism (Barua et al., 2002; Allam et al., 2013; Madani et al., 2018). Indeed, elevated amounts of circulating endothelial microparticles were found in smokers (Badrnya et al., 2014; Liu et al., 2014; Mobarrez et al., 2014).

Clinically, there is growing evidence that a subset of non-COPD smokers present with imaging evidence of microvascular pruning or constriction (Iyer et al., 2016; Saruya et al., 2016) and functional abnormalities consistent with the areas of increased ventilation-perfusion relationship (Gläser et al., 2013; Rizzi et al., 2016; Bodduluri et al., 2017). Interestingly, ventilatory inefficiency has been associated with impaired flow-mediated dilation in smokers, supporting a generalized vasculopathy (Gläser et al., 2011). Impaired ability in recruiting pulmonary vessels during exercise has been demonstrated in light smokers (Rizzi et al., 2016) or second-hand smokers (Arjomandi et al., 2012). Moreover, a large population-based study showed the presence of pulmonary artery enlargement on HRCT in smokers without COPD (Lindenmaier et al., 2016). Overall, compensatory increases in minute ventilation are likely useful to maintain alveolar ventilation and arterial blood gas homeostasis in symptomatic smokers but this might hasten dynamic mechanical constraints thereby contributing to dyspnea and exercise intolerance. (Elbehairy et al., 2016; Di Marco et al., 2017) These physiological considerations should be tempered with the observation that smokers have two to four times more panic-depression and anxiety disturbances compared to controls (Zvolensky et al., 2004; Moylan et al., 2012; Fadda et al., 2013). These abnormalities are associated with a chaotic breathing pattern and hyperventilation sindromes (Bokov et al., 2016; Bansal et al., 2018), and accordingly, contemporary models of fatigue point out to complex interactions between physiological activity and psychological state (Gruet, 2018). Hence, such complex “coordinated deadaptation”(Burtscher, 2013) in symptomatic smokers might lead to perceived fatigability (sensations about fatigue) and performance fatigability (incapacity of the neuromuscular system to meet the requirements of a given task) (Enoka and Duchateau, 2016). Thus, objective and subjective mechanisms may dynamically interact and prompt early exercise cessation in susceptible smokers.

Finally, there is limited evidence that some smokers may present with reduced inspiratory muscle strength (Formiga et al., 2018; Bostanci et al., 2019) and endurance though this is not a universal finding (Elbehairy et al., 2016). Owing to exquisitive sensitivity of the diaphragm to hypoxia (Zhu et al., 2005; Lewis and O’Halloran, 2016), low-grade inflammation (Haegens et al., 2012), and oxidative stress (Lawler and Powers, 1998; Barreiro et al., 2006), it remains possible that it might suffer the consequences of chronic smoking. During exercise, O_2_ delivery to the respiratory muscles might be impaired in some smokers – similarly to what has been demonstrated in the peripheral muscles in non-COPD smokers – at very high levels of ventilation (Kimura et al., 2007). In the above-mentioned study by Elbehairy et al. (2016), the authors found that the rib cage and accessory muscles contributed to a greater extent to meet a heightened ventilatory response to exercise in symptomatic smokers. This might constitute a useful strategy to spare a mechanically stressed diaphragm. In view of the experimental data supporting diaphragm wasting secondary to tobacco exposure, (Carlos et al., 2014; Vieira Ramos et al., 2018) increased ventilatory demands during exercise might overload the diaphragm, thereby contributing to exertional dyspnea.

## Conclusions

Multiple interrelated mechanisms may decrease the ability of smokers without COPD to face the challenges brought by physical exercise. In fact, the stress of exercise constitutes a physiologically elegant – and clinically relevant – model to expose the deleterious effects of oxidative stress, pro-inflammatory status, sustained high-circulating nicotine levels, low O_2_ content, and high carbon monoxide on human health well before they are apparent at rest. Although physical inactivity and, potentially, specific psychological traits are major confounders, there seems to exist a subset of smokers who are particularly intolerant to exertion. Complex and yet poorly understood interactions among cardiopulmonary and muscular abnormalities might underlie this specific phenotype of “symptomatic smokers on exertion.” A deeper understanding of the systemic effects of smoking in subjects who did not (yet) show evidences of devastating smoking related diseases, such as COPD and ischemic heart disease, might prove instrumental to fight their ever-growing burden.

## Author Contributions

All authors listed have made a substantial, direct and intellectual contribution to the work, and approved it for publication.

### Conflict of Interest Statement

The authors declare that the research was conducted in the absence of any commercial or financial relationships that could be construed as a potential conflict of interest.
